# Factors affecting the loss of MED12-mutated leiomyoma cells during *in vitro* growth

**DOI:** 10.18632/oncotarget.16711

**Published:** 2017-03-30

**Authors:** Jeannine Bloch, Carsten Holzmann, Dirk Koczan, Burkhard Maria Helmke, Jörn Bullerdiek

**Affiliations:** ^1^ Institute of Medical Genetics, University Rostock Medical Center, D-18057 Rostock, Germany; ^2^ Institute of Immunology, University Rostock Medical Center, D-18057 Rostock, Germany; ^3^ Institute of Pathology, Elbe Kliniken, Klinikum Stade, D-21682 Stade, Germany; ^4^ Center of Human Genetics, University of Bremen, D-28359 Bremen, Germany

**Keywords:** MED12 mutations, uterine leiomyomas, in vitro growth, model system, cell culture

## Abstract

Uterine leiomyomas (UL) are the most prevalent symptomatic human tumors at all and somatic mutations of the gene encoding mediator subcomplex 12 (*MED12*) constitute the most frequent driver mutations in UL. Recently, a rapid loss of mutated cells during *in vitro* growth of UL-derived cell cultures was reported, resulting in doubts about the benefits of UL-derived cell cultures. To evaluate if the rapid loss of *MED12*-mutated cells in UL cell cultures depends on *in vitro* passaging, we set up cell cultures from nine UL from 40–50 year old Caucasian patients with at least one UL. Cultured UL cells were investigated for loss of *MED12*-mutated cells. Genetic characterization of native tumor samples and adjacent myometrium was done by array analysis. “Aged” primary cultures without passaging were compared to cells of three subsequent passages. Comparative analyses of the mutated/non-mutated ratios between native tissue, primary cells, and cultured tumor cells revealed a clear decrease of *MED12*-mutated cells. None of the tumors showed gross alterations of the array profiles, excluding the presence of gross genomic imbalances besides the *MED12* mutations as a reason for the intertumoral variation in the loss of *MED12*-mutated cells. Albeit at a lesser rate, loss of *MED12*-mutated cells from cell cultures of UL occurs even without passaging thus indicating the requirement of soluble factors or matrix components lacking *in vitro*. Identification of these factors can help to understand the mechanisms of the growth of the most frequent type of uterine leiomyomas and to decipher novel drug targets.

## INTRODUCTION

Uterine leiomyomas (UL) are the most prevalent symptomatic human tumors at all constituting a major public health problem [1, 2].

Nevertheless, there is a surprising gap of knowledge on the pathobiology of these frequent tumors. Current data strongly suggest that particular somatic mutations of the gene encoding mediator subcomplex 12 (*MED12*) constitute the most frequent driver mutations in UL [3–7]. Mediator subcomplex 12 is a protein involved in the organisation of the transcription machinery showing a high degree of evolutionary conservation among mammalian species in particular in the region encoded by the UL-hotspot region [8]. While UL-derived cell cultures are a wide-spread model system for research into the pathogenesis and behavior of the tumors [2, 9–11] recent results challenge the usefulness of this model for the group of tumors carrying *MED12* mutations. Contrary to the expectations a rapid loss of mutated cells accompanied *in vitro* growth of UL-derived cell cultures [12]. As a rule, the mutations were not even detectable after early passages of *in vitro* growth resulting from so far unknown mechanisms. One possible explanation is a decreased growth potential resulting from *in vitro* passaging. However, an in depth understanding of the mechanisms leading to the disappearance of tumor cells *in vitro* may help to uncover basic growth requirements of *MED12*-mutated UL and as well as novel drug targets. Thus, it is of pivotal interest to see if the disappearance of mutated cells is a phenomenon linked to enzymatic treatment of cells associated with passaging or occurs independent of this artificial procedure. Accordingly, we have performed a series of experiments aimed at a comparison between cells of UL subjected to repeated passaging and those that were kept in continuous stationary primary culture.

## RESULTS

### *MED12* mutations were found in the majority of tumors initially analyzed

Initially, cell cultures from nine UL derived from five patients were set up for cell culture. The results of *MED12* mutation analyses revealed mutations of that gene in seven of these tumors (Table 1). Histologically, all seven tumors were typical UL. Of these one carried a *MED12* in-frame deletion and the others showed single-base substitutions (Supplementary Figure 1). All mutations detected were identical to those previously detected in UL and heterozygous. Accordingly, Sanger sequencing resulted in peaks corresponding to the non-mutated and mutated alleles, respectively. For further evaluation it was presumed that the “non-mutated peak” results from the normal allele of the tumor cells as well as from the two normal alleles of bystander cells not belonging to the tumor cell population. *Vice versa*, the “mutated peak” is considered to have originated exclusively from the mutated allele of the tumor cells.

**Table 1:** Type of MED12 mutation in leiomyomas with MED12 mutations included in the study

tumors investigated	age	tumor size (cm)	*MED12* status
06/1	52	7.5	c.131G>C
06/2		4.5	c.131G>C
07/1	47	1.8	c.131G>A
07/2		1.5	c.131G>A
07/3		1.0	c.126_137del12
12/1	53	1.0	c.107T>G
12/2		0.6	c.131G>A

### In most cases, a decrease of mutated cells was noted even in the primary culture

**Figure 1:**
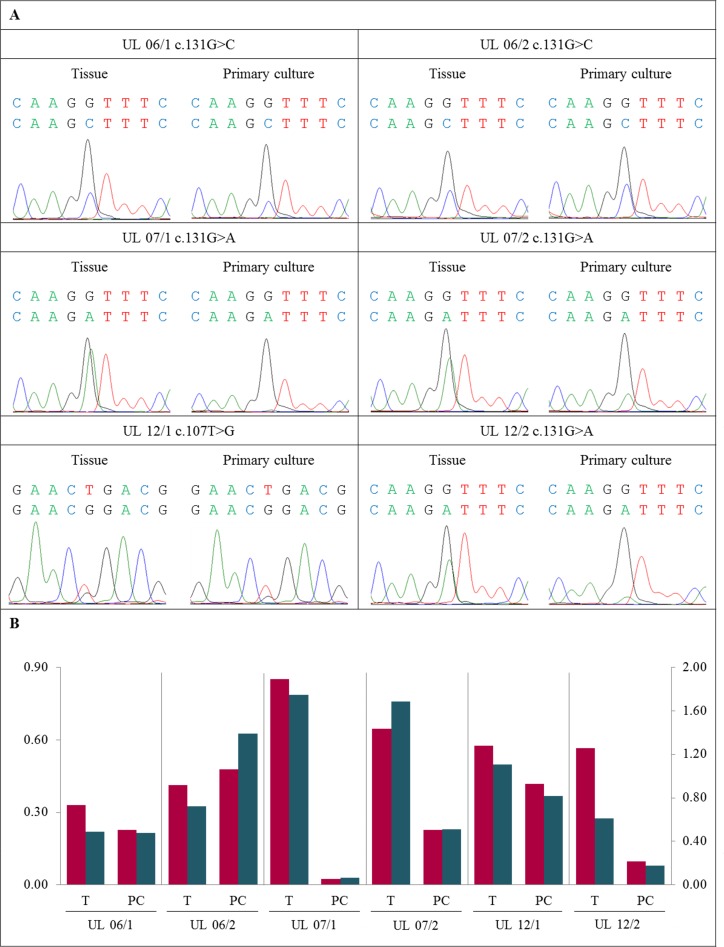
Decrease of *MED12*-mutated cells in primary cultures compared to native tissue (**A**) DNA forward sequence of three codons around the *MED12* point mutation of the native tumor tissue (T) and the corresponding primary culture (PC) displaying heterozygous mutations c.131G>C, c.131G>A or c.107T>G, respectively. In UL 06/1, UL 07/1, UL 07/2, UL 12/1 and UL 12/2 a decline of the peak indicating the mutated allele was detected in PC. In case UL 06/2 a moderate increase of the mutated peak was noted. (**B**) Quantification of mutated to non-mutated allele of *MED12* mutation mainly shows a decrease of *MED12*-mutated cells in PC compared to corresponding T. The quotients of forward sequencing direction (red columns) were shown on primary axis and of reverse (blue columns) on secondary axis.

First, six of these cases (UL 06/1, UL 06/2, UL 07/1, UL 07/2, UL 12/1, and UL 12/2) were used for a comparative analysis of the peak ratios between native tissue and primary cultures. In case UL 07/3, cells of the primary culture were not available for analysis. Compared to the native tissue the relative height of peaks corresponding to the mutated allele had decreased in the primary cultures of 5/6 tumors (UL 06/1, UL 07/1, UL 07/2, UL 12/1, and UL 12/2) investigated. Nevertheless, a strong variation of the decrease was noted that ranged between an almost complete disappearance of the mutated peak seen in UL 07/1, a huge loss of 67% to 77% in UL 07/2 and UL 12/2, and a slight decrease of only 17% to 27% observed in UL 06/1 and UL 12/1, respectively (Figure 1 and Supplementary Figure 3). In the remaining case (UL 06/2), a moderate increase of the ratio was noted in the primary culture.

### Further disappearance of mutated cells does not require passaging but also occurred in “aging” primary cultures

**Figure 2:**
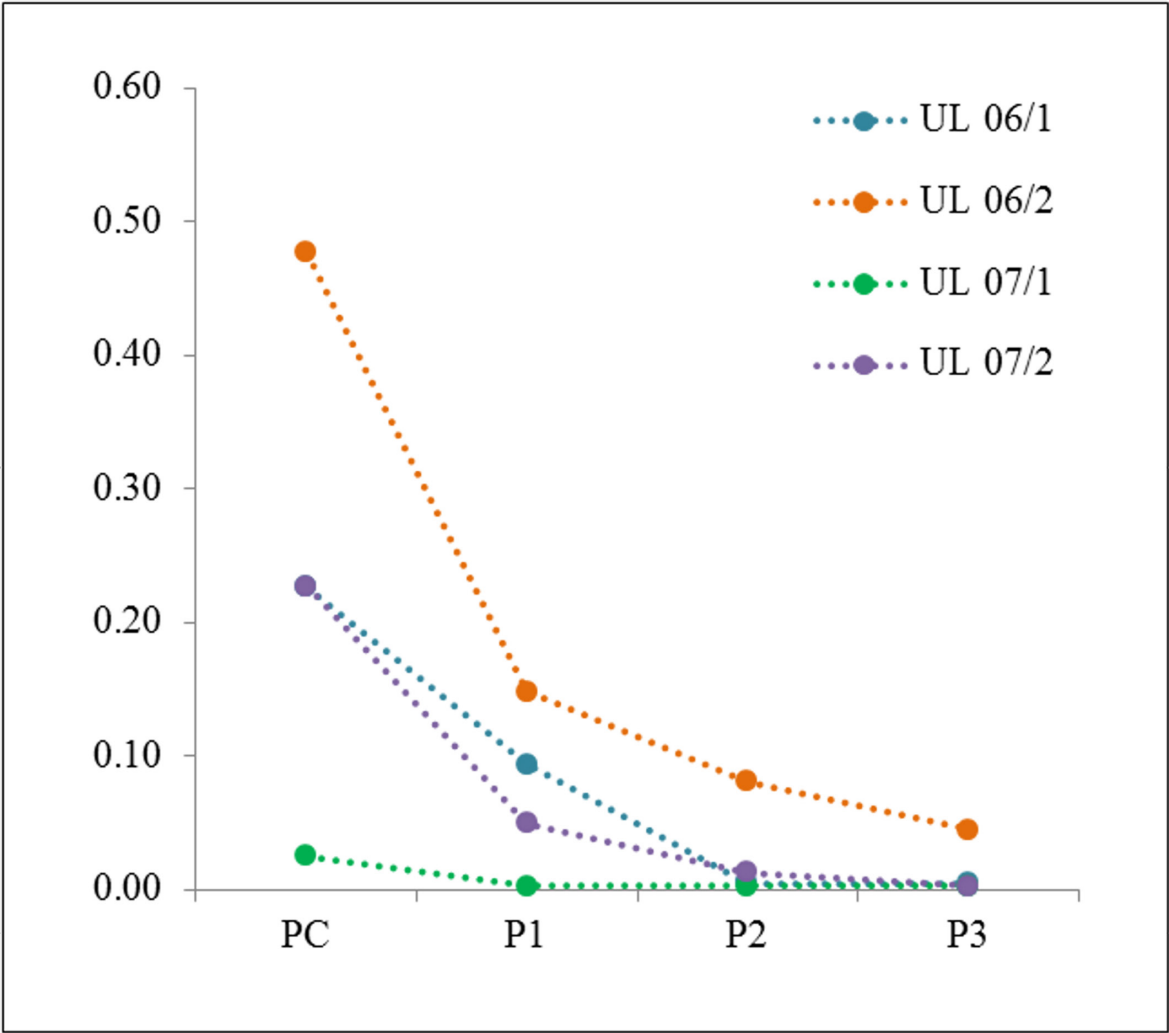
Disappearance of cells with *MED12* mutation during *in vitro* passaging In all UL investigated a loss of mutated/non-mutated peak ratio was noted in the first passage (P1) compared to the corresponding primary culture (PC). In the following passages (P2 or P3) the loss further declines to an almost complete loss of mutated cells, except UL 06/2. Data resulting from DNA forward sequencing.

As a next step, we were interested in monitoring changes of the frequency of the *MED12* mutated cells during *in vitro* propagation. Therefore, we passaged the “young” primary cultures of five tumors (UL 06/1, UL 06/2, UL 07/1, UL 07/2, and UL 07/3) three times followed by sequencing. In all cases continuous passaging was accompanied by a further reduction of the peak ratio compared to the native tissue (5/5 cases) as well as to the primary culture (4/4 cases, no data of the primary culture were available for UL 07/3) (Figure 2). In the first passages the decline ranged between 60% and 80% compared to the corresponding primary culture. In UL 06/1, UL 06/2 and UL 07/2 the mutation was clearly detectable in the first passage but rapidly disappeared in passage 2 and 3. In contrast, UL 07/1 showed an almost complete loss of the mutated peak already in the first passage. As to further passaging the *MED12* mutation was almost undetectable in three of the cases whereas it remained clearly visible until passage 3 in UL 06/2. Moreover, the number of passages where mutated cells were still detectable seems to depend on their frequency in the primary culture.

**Figure 3:**
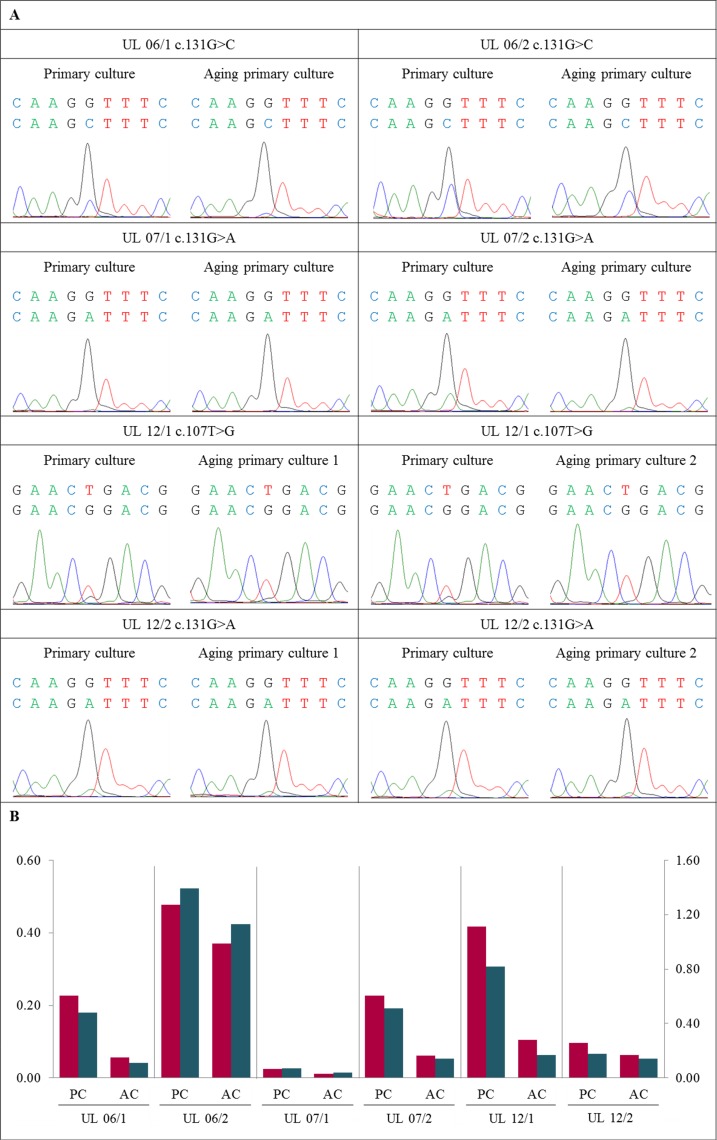
Further disappearance of *MED12*-mutated cells also occurred in “aging” primary cultures (**A**) DNA forward sequence of three codons around the *MED12* point mutation of the primary cell culture (PC) and the corresponding “aging” primary cultures (AC) displaying a heterozygous mutation c.131G>C, c.131G>A or c.107T>G. In all UL investigated a decline of the “mutated peak” was detected in AC compared to matching PC. (**B**) Quantification of mutated to non-mutated allele of *MED12* mutation shows a decrease of *MED12*-mutated alleles in AC compared to corresponding PC. The quotients of forward sequencing direction (red) were shown on primary axis and of reverse (blue) on secondary axis.

Next we were interested to see if a simple “aging” of the confluent primary cultures leads to a decrease of the mutated cells. Accordingly, primary cultures of six tumors were kept unpassaged after they had reached confluency. During that time, medium was changed every 2^nd^ or 3^rd^ day until they were harvested. Compared to the “young” primary cultures “aging” of the primary culture resulted in a reduced mutated/non-mutated peak ratio in all six tumors (UL 06/1, UL 06/2, UL 07/1, UL 07/2, UL 12/1, and UL 12/2) (Figure 3 and Supplementary Figure 4). In two tumors (UL 12/1 and UL 12/2) “aging” primary cultures were harvested and analyzed at two different times of *in vitro* growth. The cells were harvested one and two weeks after “young” primary cultures. In general, both points in time revealed a decline in mutated to non-mutated peak compared to the “young” primary cultures. While in UL 12/1 a loss of 60% and 80%, respectively, was noted in both “aging” primary cultures, in UL 12/2 the ratio of mutated/non-mutated peaks was decreased to 30% and remained nearly unchanged. However, in neither of the cultures the disappearance of mutated cells was accompanied by clear changes of cellular morphology.

### Passaging of the cells accelerated the loss of mutated cells

**Figure 4:**
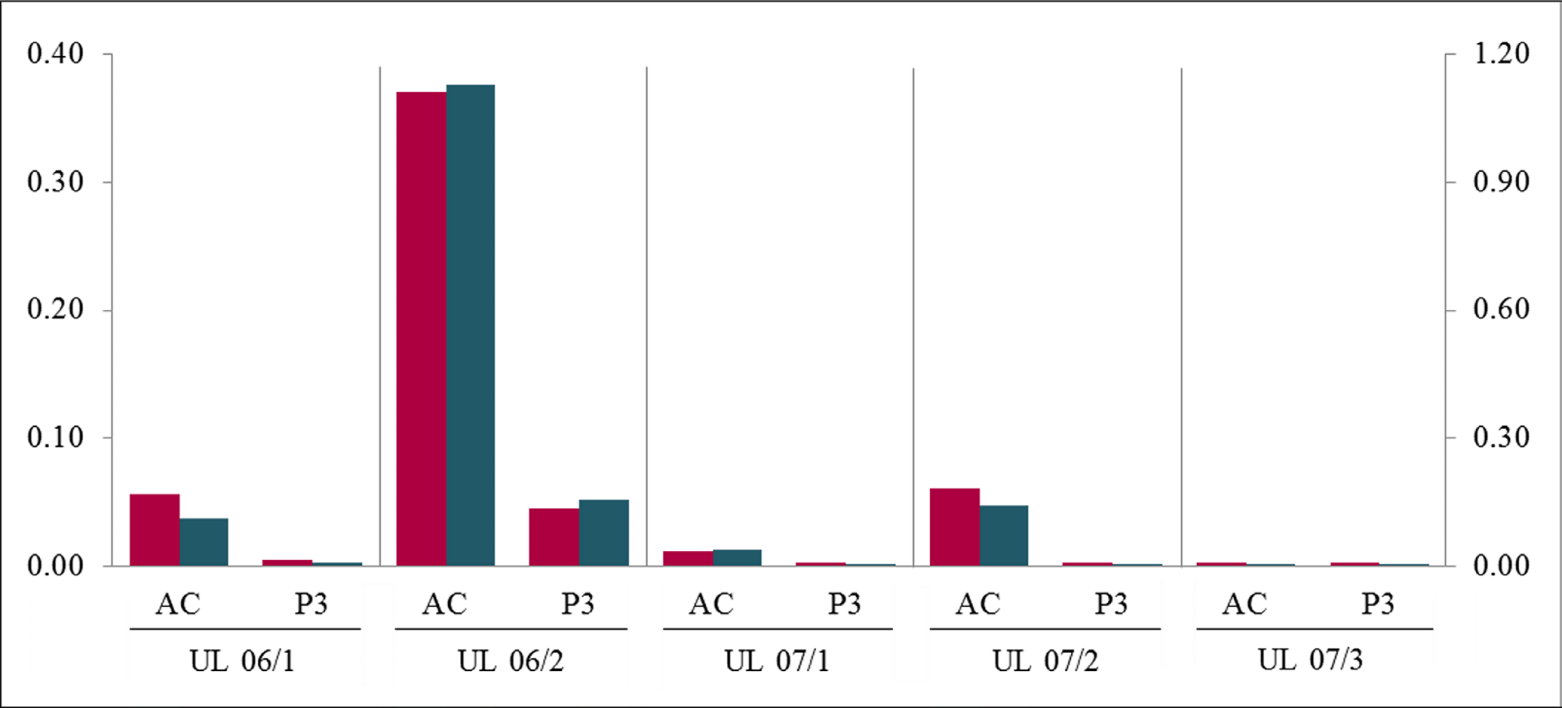
Passaging of cells accelerated the loss of *MED12*-mutated cells compared to simple “aging” In all UL the quantification of mutated to non-mutated allele of *MED12* mutation shows a decrease of *MED12*-mutated alleles in passage 3 (P3) compared to the corresponding “aging” primary culture (AC) harvested at the same time period. The quotients of forward sequencing direction (red) were shown on primary axis and of reverse (blue) on secondary axis.

We hypothesized that the procedures necessary for passaging did not cause but may accelerate the loss of mutated cells from the cultures. To validate this assumption we have compared the loss in “aging” primary cultures with the corresponding cell cultures that had been passaged within the same interval of time. For this comparison, five pairs of 3^rd^ passage cells along with their matching unpassaged primary cultures of the same “age” were used. The results show that in all five cell cultures investigated the loss occurred indeed more rapidly when cells are passaged. While the loss strongly varied between the tumors, in all these pairs the amount of mutated cells in the “aged” cultures exceeded that seen in the 3^rd^passage cells of the same “age” (Figure 4). Furthermore, in 3/4 cultures the ratio in the “aged” cultures exceeded that observed in the cells of the 1st passage.

### The different rates of loss of mutated cells did not correlate with the presence of genomic imbalances in addition to the *MED12* driver mutations

While the decrease of mutated cells *in vitro* strongly varied among the different tumors investigated no obvious explanation for these differences was apparent. *MED12* mutations in UL are known to coincide often with other genetic aberrations as e.g. deletions of part of the long arm of chromosome 7, rearrangements of the *HMGA1* locus at 6p21 or trisomy 12 in a considerable percentage of cases [4]. These alterations might influence the “*in vitro* behavior” of *MED12*-mutated cells. Thus, aimed at the question whether or not the differences are due to secondary genomic gains and losses in addition to the *MED12* mutations we used CNV arrays for the analysis of the native tumor samples along with their matching myometrium on four UL (UL 06/1, UL 06/2, UL 07/1, and UL 07/2) with *MED12* mutations. In none of the tumors investigated relevant alterations of the CNV profiles became apparent when comparing the leiomyomas with the corresponding normal tissue (Supplementary Figure 6). Thus, the reasons for the different kinetics of the loss of mutated cells still remain to be elucidated.

### Loss of mutated cells from the “aging” culture correlates with a detachment of cells from the monolayer

**Figure 5:**
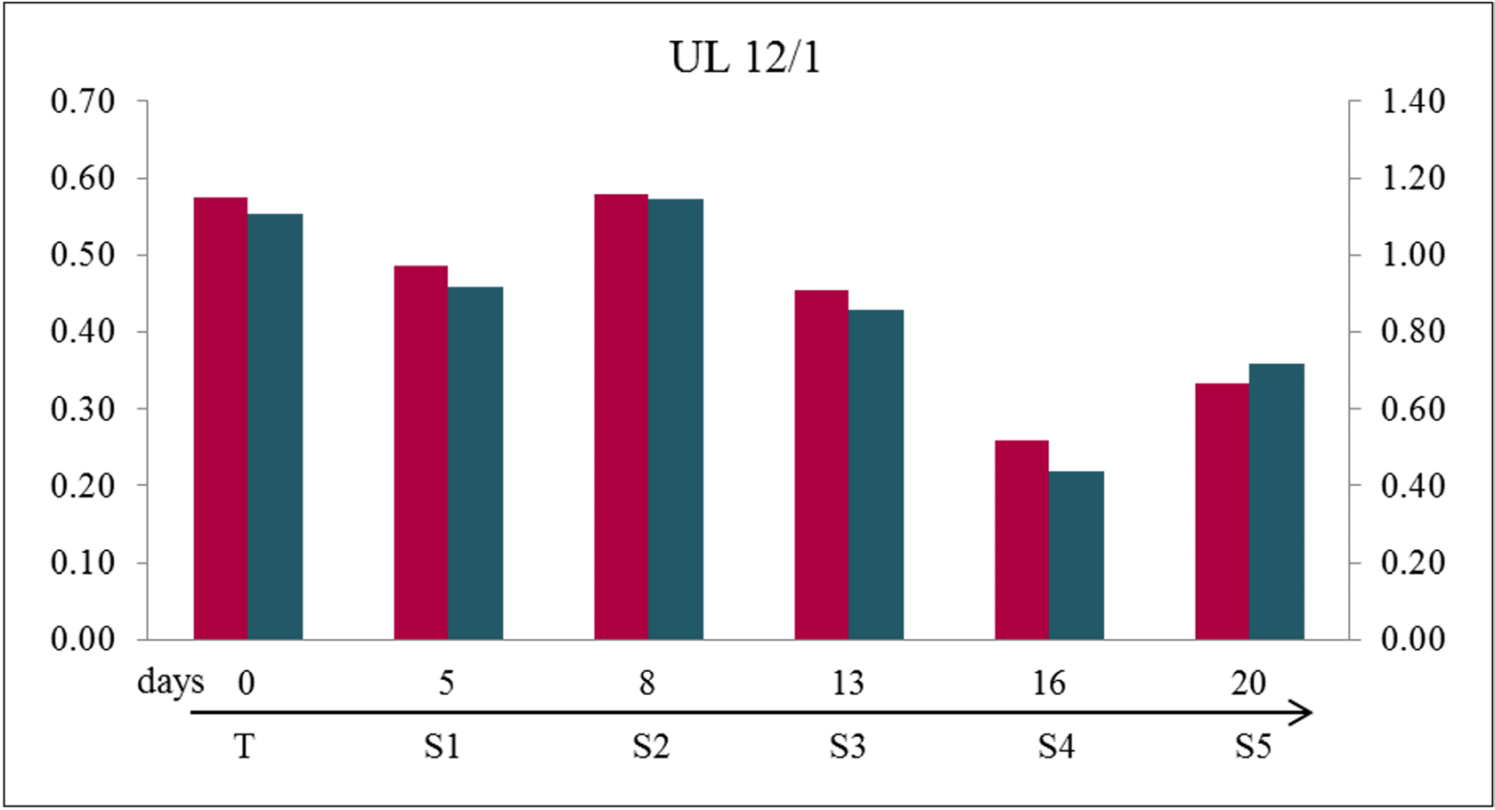
Detachment of cells from the monolayer during “aging” could cause the loss of mutated cells during *in vitro* culturing Quantification of mutated to non-mutated allele of *MED12* mutation of cells in the supernatant (S1 to S5) revealed a large mutated/non-mutated peak ratio comparable to native tumor tissue (T) indicating a large amount of mutated cells in the supernatant. The numbers represent the days where the supernatants were obtained starting with set up the cell culture using the example of UL 12/1. The quotients of forward sequencing direction (red) were shown on primary axis and of reverse (blue) on secondary axis.

**Figure 6:**
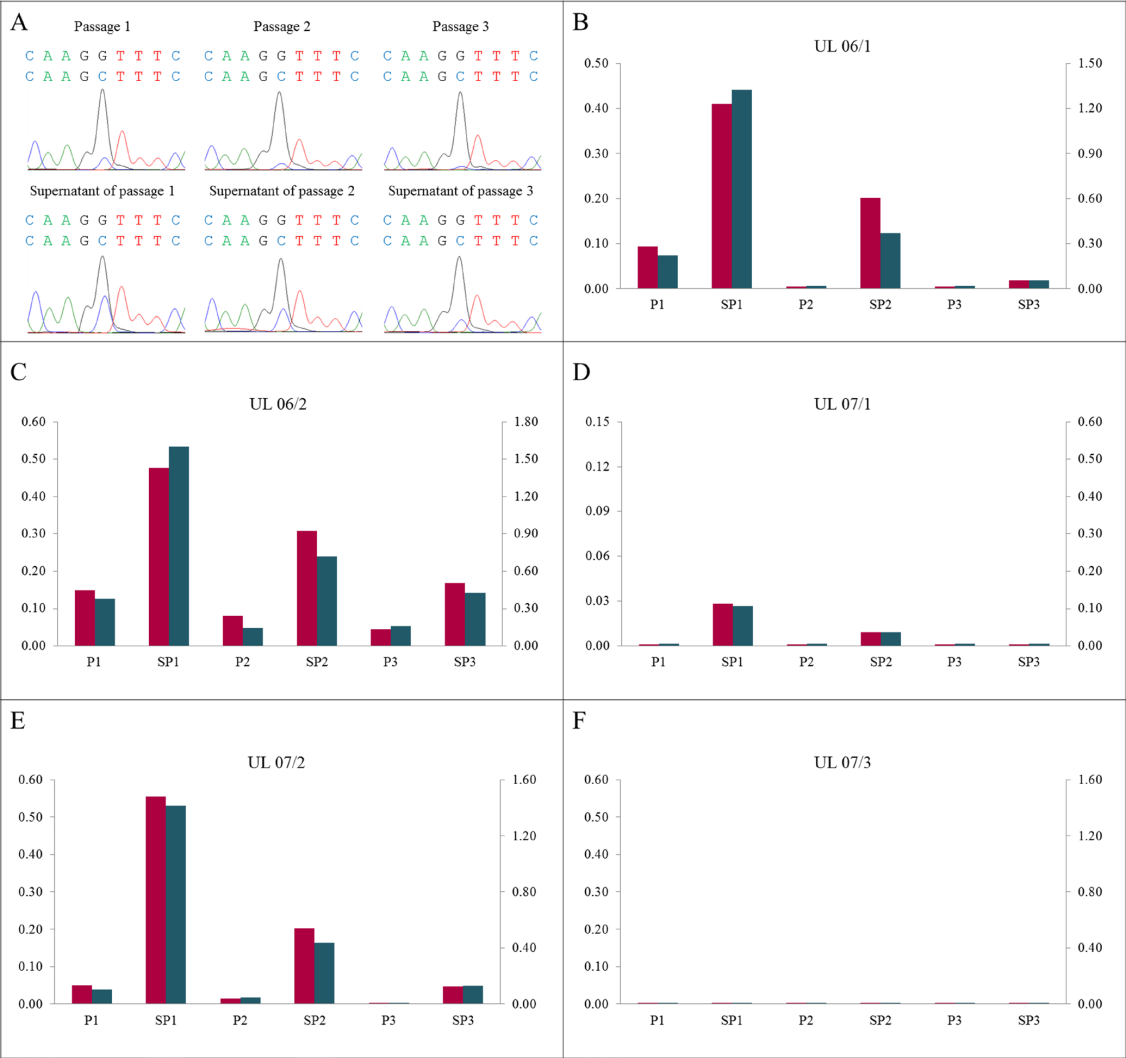
A high proportion of mutated cells in the supernatant of corresponding passages (**A**) DNA forward sequences of cells in the supernatants (SP1 to SP3) show a higher “mutated peak” compared to the corresponding passages (P1 to P3) using the example of UL 06/2. (**B**–**F**) The mutated/non-mutated peak ratios in the supernatants clearly exceeded the ratios in the corresponding passages in all UL investigated. In increasing numbers of passages and supernatants the ratio decreased. The quotients of forward sequencing direction (red) were shown on primary axis and of reverse (blue) on secondary axis.

As to the loss of *MED12*-mutated cells it can be assumed that the mutated cells become detached from the surface of the culture vessels. Thus, one would expect a high amount of mutated cells in the supernatant. To address this question, we have isolated and sequenced DNA from cells in the supernatant of unpassaged cultures of two UL (UL 12/1 and UL 12/2) and compared with their matching adherent cells. The results confirmed the assumption that mutated cells are lost from the monolayer by their detachment since the amount of mutated cells in the supernatant clearly exceeded that found in the corresponding monolayer (Figure 5 and Supplementary Figure 5). Simultaneously, we analyzed four UL (UL 06/1, UL 06/2, UL 07/1, and UL 07/2) and compared the DNA from the cells in the passages with their matching cells in the supernatants directly obtained after passaging. Consistent with our experience a huge amount of mutated cells was observed in the supernatant of the first passage (Figure 6). Increasing the number of passages revealed a decline of the “mutated” peak in the corresponding supernatant which, however, always remained higher than in the correlating passaged cells. Even if no evidence of mutated cells in the passaged culture was noted, they were still detectable in the corresponding supernatant. The results indicate that the mutated cells are also lost from the cell cultures due to their problems of re-attachment after passaging.

## DISCUSSION

Despite novel approaches to shrink UL and to improve tumor related bleeding [13,14] still unmet medical needs remain in the treatment of these tumors. Factors influencing the growth of UL are of high interest and often have been studied using cells grown *in vitro* (e.g. see [15–17]) making *in vitro* cultures derived from leiomyomas a widespread model system for research into biology and treatment of UL (for review see [18]).

As to their molecular pathogenesis, a number of recurrent genetic alterations confined to the ULs but not present in their surrounding myometrial tissue have been identified. For example, certain types of chromosomal deletions and translocations are often detected in UL. Among these, translocations involving chromosomal bands 12q14-15 and deletions of part of the long arm of chromosome 7 are most frequently seen and in the former case the gene encoding HMGA2, a member of the family of high mobility group proteins, has been identified as the molecular target of the alteration which becomes drastically up-regulated due to the chromosomal rearrangement [19]. Nevertheless, the majority of UL do not show any chromosomal alterations but mutations of the gene encoding Mediator subcomplex 12 (*MED12*), a protein involved in the organization of the transcription machinery. In most of these cases only a singly base exchange can be detected but small in-frame deletions and duplications do occur as well. This latter type of mutation occasionally can co-exist with chromosomal alterations except for those targeting *HMGA2* [3–7] and clearly represents the most common type of driver mutations in UL. *In vitro* cells with this type of driver mutation recently were shown to coincide with a strictly reduced growth capacity *in vitro*, leading to their rapid disappearance in cell culture [12, 20]. In contrast, other cells from the samples not carrying these mutations of presumed non-tumorigenic origin as well as from tumors carrying *HMGA2* rearrangements can survive much longer [12, 20]. The loss of *MED12*-mutated cells even during early phases of *in vitro* culturing is a phenomenon challenging many previous data obtained using cell cultures as a model system for research into the biology of UL. While it is reasonable to postulate that only the mutated cells represent the tumor cell population, the mechanisms underlying the observed loss of tumor cells are not understood yet. On the other hand, the loss of tumor cells, at least in part, may explain the rapid loss of estrogen receptors and, albeit at a lower rate, loss of progesterone receptors, repeatedly described for UL cultures based on a decrease of mRNA as well as proteins [9, 21]. This loss may, at least in part, result from the loss of positive cells from the cultures rather than from the transcriptional downregulation of the corresponding genes as previously assumed. Also, it may explain the gradual loss of cells with some types of cytogenetic alterations as e.g. deletions of the long arm of chromosome 7 known not rarely to co-exist with *MED12* mutations which has been observed in cultures obtained from UL (Sabine Bartnitzke, personal communication).

Herein, we were able to show that the treatment of cells during passaging, while accelerating their loss, does not constitute the main reason for disappearing of the mutated cells because the loss even occurs from stationary “aging” cultures. There is no obvious explanation for the strongly varying rate of loss *in vitro* but among other possible reasons the type of *MED12* mutation may play a role. Nevertheless, also in general there is no explanation for the loss of mutated cells but from our data presented herein it can be concluded that comparably “simple” explanations like e.g. a higher sensitivity of the tumor cells against trypsin treatment do not apply. Basically, two groups of explanations seem plausible.

First, it is reasonable to assume that certain soluble environmental factors not available under *in vitro* conditions used for the cell cultures are required for the survival of the tumor cells. Among these critical factors lacking *in vitro* may be growth factors and hormones. For future studies it may be worth to re-address this question by analyzing the decrease of *MED12*-mutated cells depending on different culture media including those supplemented with hormones.

Secondly, the *in vitro* growth could depend on rather “physical” factors as e.g. the presence of extracellular matrix (ECM). Interestingly, the relevance of ECM in the growth of UL had been a matter of numerous studies and therapeutical approaches targeting ECM have been proposed repeatedly (e.g. [22–25]). Accordingly, future studies should address the possible influence of ECM presented *in vitro* and of three-dimensional cell cultures on the disappearance of *MED12*-mutated cells.

As to the results of the present study we cannot decide if the main reason for the disappearance of the tumor cells is the lack of an appropriate matrix or of growth factors supporting cellular proliferation. On the other hand, growth of non *MED12*-mutated bystander cells does clearly not depend on these requirements because a rapid overgrowth of these latter cells can be noted. In general, the relative amount of non-mutated cells likely indicates that these cells are not solely derived from the tumor vasculature but represent a considerable amount of the “leiomyoma-stroma” as well. Cell cultures thus represent an interesting source to investigate the growth balance between tumor cells and non-tumorigenic cells and its disturbances, respectively because they provide an interesting system to identify factors that selectively support the growth of tumor cells. In turn, these factors may be of relevance for *in vivo* growth thus representing possible therapeutic targets. Furthermore, three-dimensional cell cultures [22, 26, 27] may become a model system suitable to replace monolayer cultures if these latter type of culturing turns out better to support the growth of *MED12*-mutated cells.

While the reduced ability of the cells will therefore lead to a re-evaluation of many data obtained in the past it, at the same time, will open novel experimental opportunities and designs. However, the results of the present study show a strong variation in the rate of loss of *MED12* mutated cells which at this time has no straightforward explanation like the presence of additional genetic abnormalities as e.g. deletions of the long arm of chromosome 7 or trisomy 12 which are known to accompany *MED12* mutations in a considerable percentage of cases. *Vice versa*, for further experiments it should be determined if there is only an intertumoral variation or if variation can be seen also between different samples from the same tumor. In any case it indicates that investigating large series of samples will be necessary to identify factors contributing to a prolonged survival of *MED12*-mutated cells *in vitro*. As to further experiments on larger series it should be noted that obviously not cells from all types of UL do require these factors essential for the growth of the *MED12*-mutated cells *in vitro*. UL harboring *HMGA2* rearrangements leading to its strong upregulation [12, 20] constitute the second largest genetic subgroup of UL the cells of which can survive for many passages under the normal culture conditions [12, 20]. Also, for further experiments aimed at supplementing various factors, studies on the clonality of the cells should accompany mutation analyses considering recent results obtained on these lesions [28].

## MATERIALS AND METHODS

### Tumor samples

Tumor samples were obtained from hysterectomy specimens immediately after surgery. All samples were taken by an experienced pathologist in order to exclude possible contamination with myometrial tissue. While one piece of each tumor was fixed in paraffin for subsequent histologic examination and one piece was snap frozen in liquid nitrogen, the remaining third was stored in sterile Hank's solution supplemented with antibiotics (500 IU/ml penicillin, 500 μg/ml streptomycin) for cell culturing. Informed written consent was obtained from all patients for genetic characterization of their tumors and use in the present study, respectively, which had been approved by the ethics committee of Ärztekammer Bremen.

### Cell cultures

From tumor samples stored in Hank's solution, cell cultures were set up as described recently [29]. In brief, the tissue samples were minced into small pieces and treated with collagenase (200 U) for 4.5 to 6 h. Dissociated cells were centrifuged and resuspended in culture medium (RPMI 1640 supplemented with 20% FCS, 100 IU/ml penicillin and 100 μg/ml streptomycin). The resulting cell suspension of each tumor was transferred into three cell culture flasks and incubated at 37°C and 5% CO_2_. Cultured cells were enzymatically harvested as “young” primary culture at confluency or passaged using trypsin (0.05%) with a 1:2 split ratio when reaching 80% confluency. “Aging” primary cultures were not passaged but continuously supplied with medium after confluency.

### DNA isolation

DNA from fresh frozen tissue samples and cell cultures was isolated using the QIAamp DNA Mini Kit (Qiagen, Hilden, Germany) according to manufacturer's instructions. Similarly, DNA from cells obtained from the supernatant of cell cultures was processed using the QIAamp DNA Micro Kit (Qiagen). The concentration of genomic DNA was determined by NanoDrop2000 (PeqLab, Erlangen, Germany).

### *MED12* amplification

For PCR amplification, 200 to 250 ng of genomic template DNA were used. PCR was performed as follows: 1 cycle at 95°C for 15 min followed by 35 cycles of 94°C for 0.5 min, 59°C for 0.5 min, 72°C for 0.5 min, and a final extension at 72°C for 6 min. Primers used to amplify *MED12* exon2 of the genomic template DNA were 5′-GCCCTTTCACCTTGTTCCTT-3′ (forward) and 5′-TGTCCCTATAAGTCTTCCCAACC-3′ (reverse), recently described by Mäkinen *et al*. [3]. Subsequently, PCR-products were electrophoretic separated using a 2% agarose gel and the desired PCR fragments were extracted by a QIAquick Gel Extraction Kit (Qiagen) according to manufacturer's instructions or directly purified using the QIAquick PCR Purification Kit (Qiagen).

### Sequencing

DNA sequencing of the purified PCR products was performed using the GenomeLab DTCS-Quick Start Kit (Beckmann Coulter, Krefeld, Germany) and the GenomeLab GeXP Genetic Analysis System CEQ 8800 (Beckman Coulter) according to manufacturer's instructions. For Sanger sequencing, 20 ng of the PCR product was used as template for both, forward and reverse sequencing. DNA sequencing data were analyzed using the software of Beckman Coulter Instrument (version 10.2) and BioEdit Sequence Alignment Editor (version 7.2.5, Tom Hall, USA). The sequence of NC_000023.11, NCBI, was used as reference. DNA sequencing data from matching myometria were used as controls.

### Quantification of mutated versus wild-type alleles

In case of *MED12* mutation, the electropherogram obtained by Sanger sequencing resulted in two peaks corresponding to the non-mutated and mutated allele at point mutation site in *MED12* exon 2. To determine the ratio of mutated versus wild-type alleles, the maximum fluorescence intensity of corresponding peaks was calculated from the respective BioEdit data. The resulting quotients were shown as columns for forward (primary axis) as well as reverse (secondary axis) sequencing direction for each sample. An example is shown in Supplementary Figure 2. In the absence of a detectable mutated peak the ratio was set to 0.001.

### CNV array

CNV (copy number variation) analysis was performed using premade CytoScan HD Arrays (Affymetrix, Santa Clara, CA) consisting more than 2.4 million markers for copy number alterations and approximately 750,000 markers for single nucleotide polymorphisms (SNPs). Enriched gene coverage results in a marker-base ratio coverage of 1/384 for ISCA, 1/553 for cancer genes, 1/486 for X-chromosomal genes, and 1/659 for 12,000 OMIM genes. The manufacturer's instructions were followed for labelling of 300 ng DNA, and hybridization. After staining and washing using a GeneChip Fluidics Station 450 (Affymetrix) the arrays were scanned by an Affymetrix 3000 7G scanner and analyzed through the Affymetrix Chromosome Analysis Suite (ChAS) software (ChAS analysis files for the CytoScan® HD Array version NA33). Numbering of map positions was based on hg19 (NCBI Build 37 reference sequence). Filter settings of copy number changes across the genome were ≥ 25 kbp and marker count ≥ 30.

## SUPPLEMENTARY MATERIALS FIGURES AND TABLES


