# Supplementary material for: Factors affecting the loss of MED12-mutated leiomyoma cells during in vitro growth
Source: Oncotarget. 2017 Mar 30;8(21):34762–72. doi: 10.18632/oncotarget.16711 (PMC5471009; doi:10.18632/oncotarget.16711)
Supplement: Supplementary file 1 [file oncotarget-08-34762-s001.pdf]

Factors affecting the loss of *MED12*-mutated leiomyoma cells during *in vitro* growth

Supplementary Materials

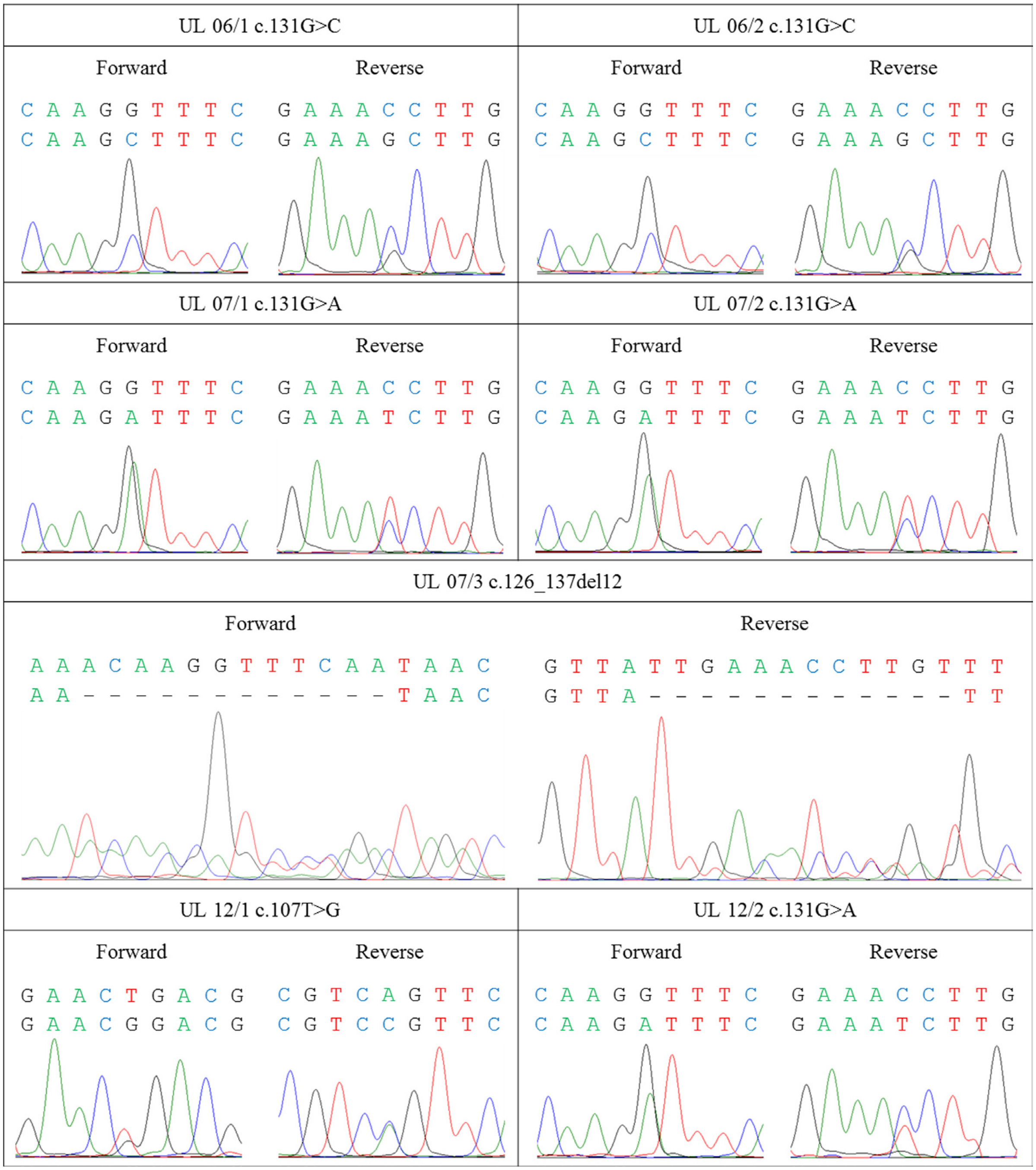

Supplementary Figure 1: Seven tumors display a heterozygous mutation in *MED12*. DNA forward and reverse sequence of three codons around the *MED12* mutation of the native tumor tissue display a heterozygous mutation c.131G>C, c.131G>A or c.107T>G and a 12 bp deletion (c.126_137).

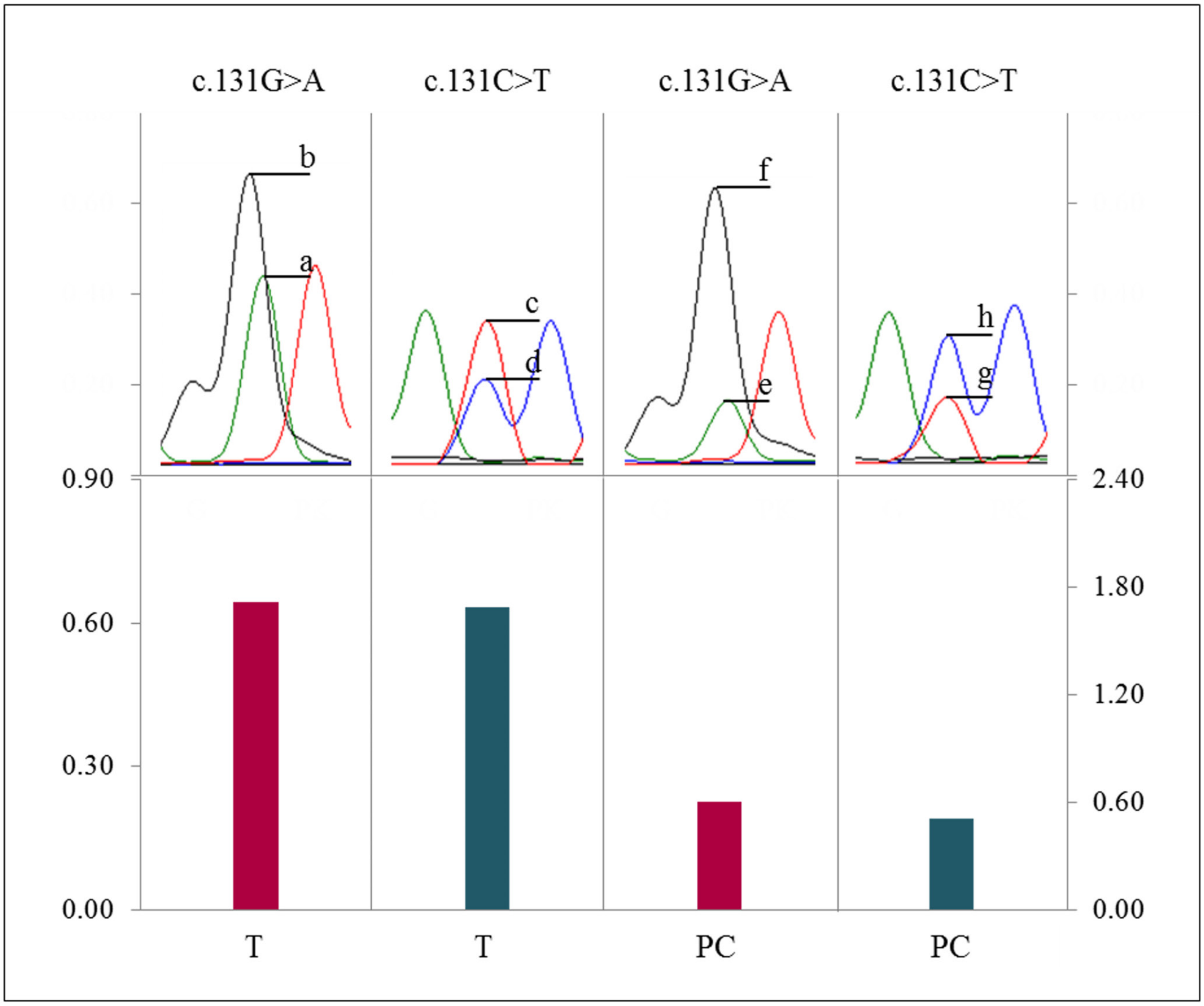

Supplementary Figure 2: Quantification of mutated to wild-type allele based on the electropherogram. The maximum fluorescence intensity of corresponding mutated (**a**, **c**, **e**, **g**) and non-mutated (**b**, **d**, **f**, **h**) peaks determined from the electropherograms is used to calculate the ratio. The resulting quotients are shown as columns for forward (red columns, primary axis) as well as reverse (blue columns, secondary axis) sequencing direction for each sample. DNA forward sequence of one codon around the *MED12* mutation c.131G>A of the native tumor tissue (T) and the corresponding primary culture (PC) is used to illustrate the quantification.

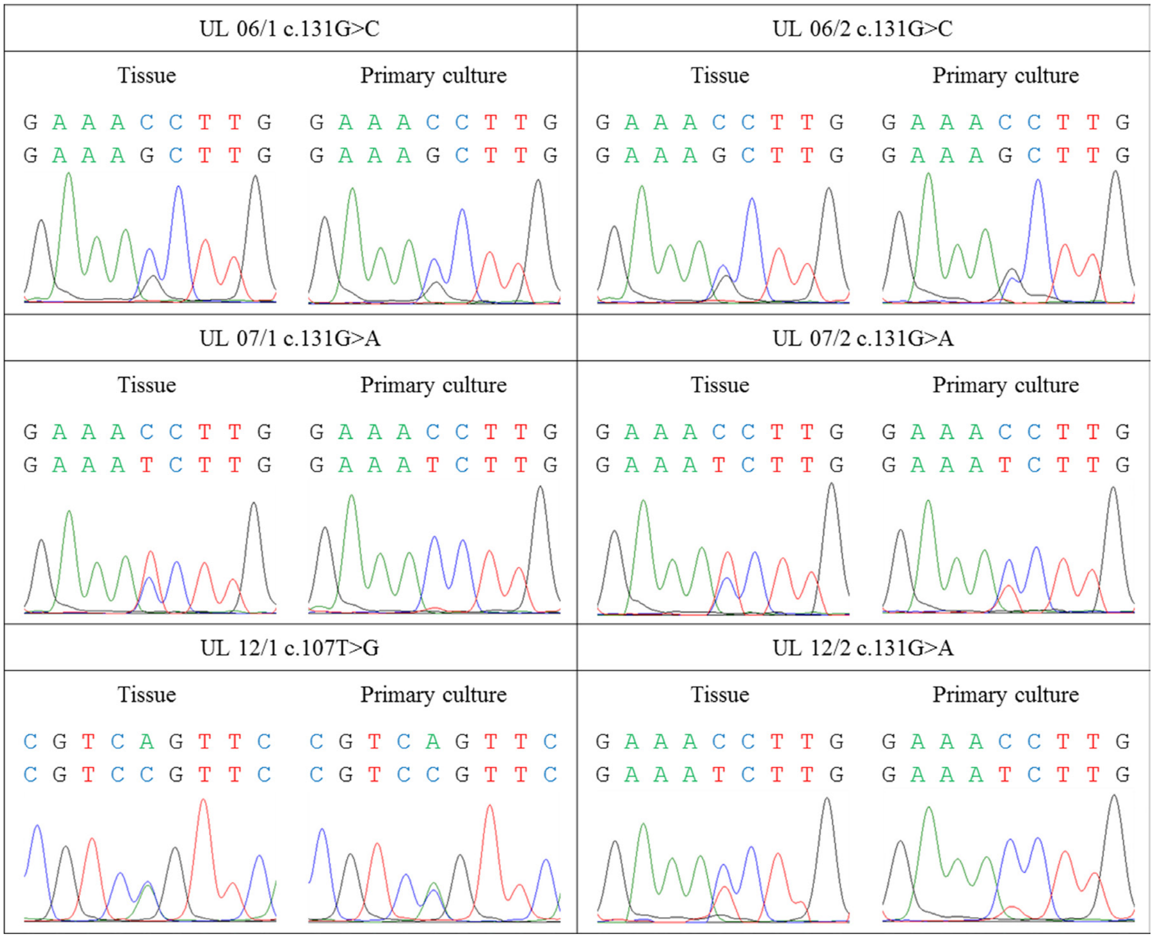

Supplementary Figure 3: Decline of *MED12*-mutated cells in primary culture compared to tissue (reverse). DNA reverse sequence of three codons around the *MED12* mutation of the native tumor tissue (T) and the corresponding primary culture (PC) displaying a heterozygous mutation c.131G>C, c.131G>A or c.107T>G. In UL 06/1, UL 07/1, UL 07/2, UL 12/1 and UL 12/2 a decline of the peak corresponding to the mutated allele was detected in PC. In case UL 06/2 a moderate increase of the mutated peak was noted.

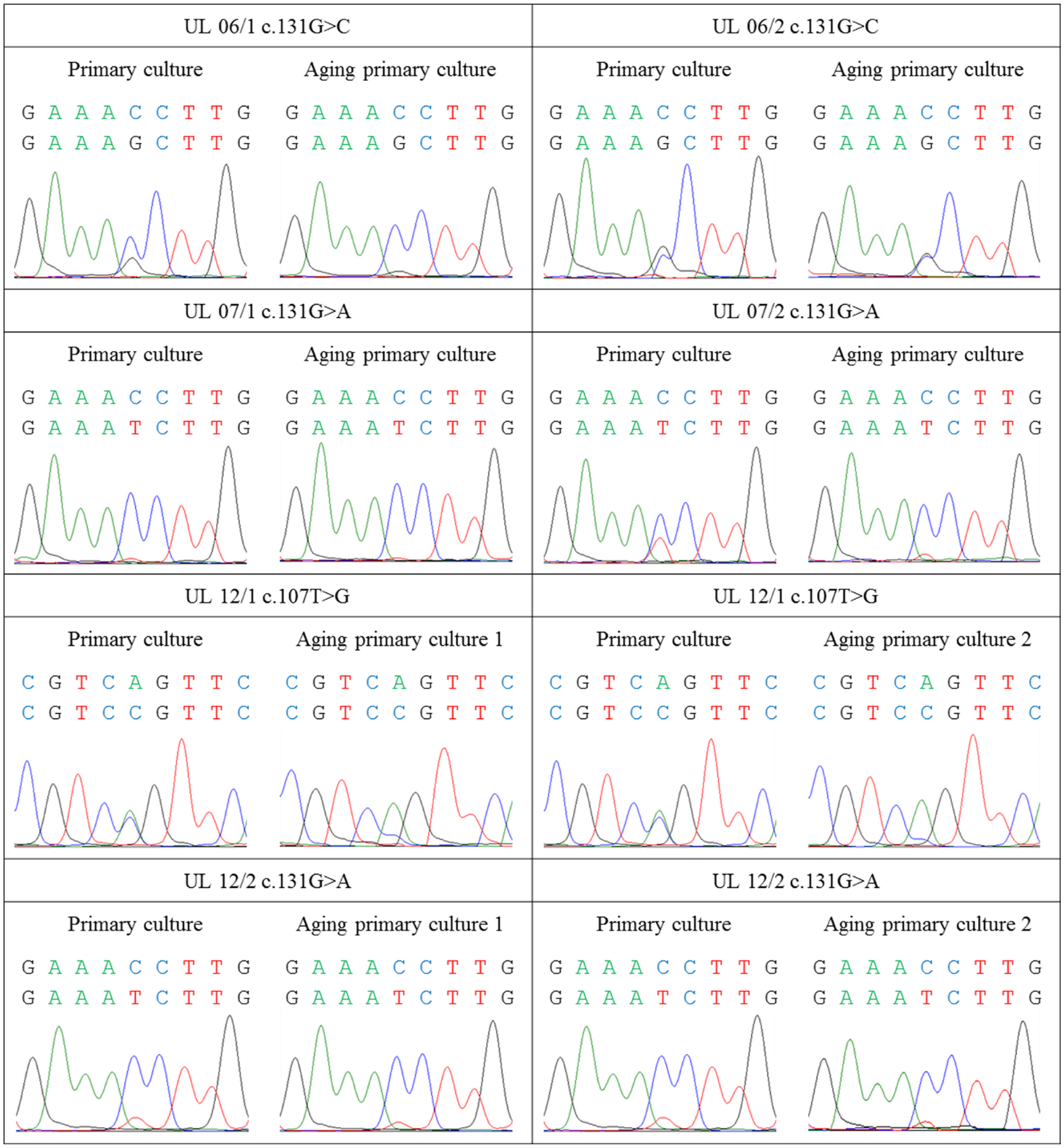

Supplementary Figure 4: Decrease of *MED12*-mutated cells in "aging" primary culture compared to "young" primary culture (reverse). DNA reverse sequence of three codons around the *MED12* mutation of the primary cell culture (PC) and the corresponding "aging" primary cultures (AC) displaying a heterozygous mutation c.131G>C, c.131G>A or c.107T>G. In all UL investigated a decline of the "mutated peak" was detected in AC compared to matching PC.

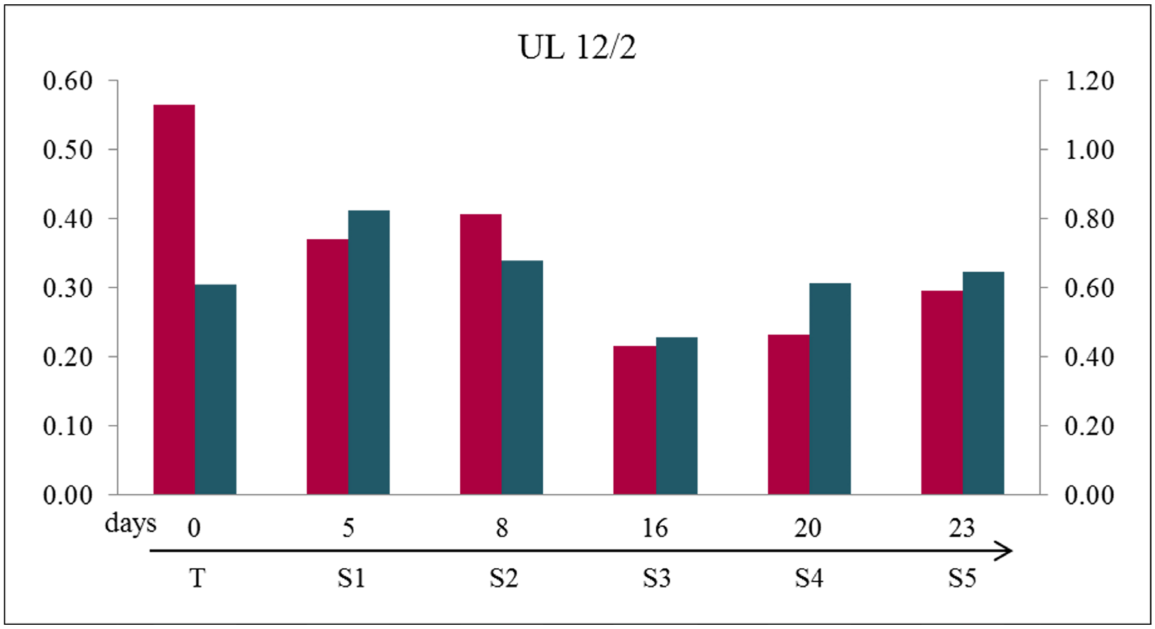

Supplementary Figure 5: A considerable amount of cells in the supernatant of "aged" primary cultures indicates a detachment of mutated cells from the monolayer. Quantification of mutated to non-mutated allele of *MED12* mutation of cells in the supernatant (S1 to S5) revealed a large mutated/non-mutated peak ratio comparable to native tumor tissue (T) indicating a large amount of mutated cells in the supernatant. The numbers represent the days where the supernatants were obtained starting with set up the cell culture using the example of UL 12/2. The quotients of forward sequencing direction (red columns) are shown on primary axis and of reverse (blue columns) on secondary axis.

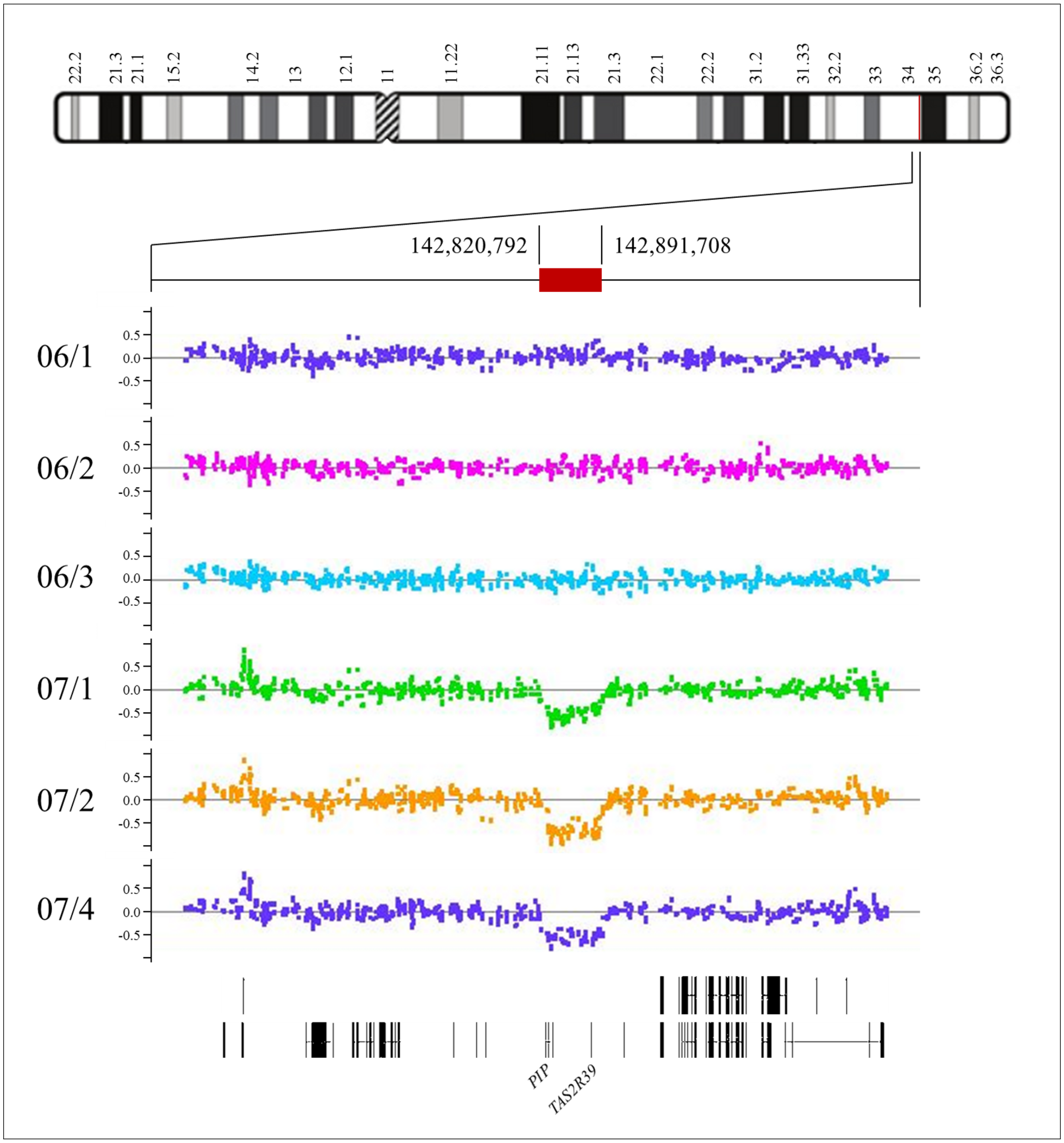

Supplementary Figure 6: The normal CNV profile of tumors compared to their matching myometria using the example of chromosome 7q34. The CNV profile of one's patients UL (UL 06/1, UL 06/2 or UL 07/1, UL 07/2) shows no differences among each other compared to the corresponding myometrium (UL 06/3 or UL 07/4). Unlike ULs 06, ULs 07 reveal a deletion of about 71 kbp which involves DGV structural variants and includes two genes (*PIP*, *TAS2R39*).

Supplementary Table 1A–1D: Data of analyzed cells of UL investigated

Supplementary Table 1A:

ratio		forward	reverse	T vs. PC	forward [%]	reverse [%]	mean [%]	SD [%]
UL 06/1	T	0.3298	0.4894	remained	68.7	98.0	83.3	20.7
	PC	0.2264	0.4795	decreased	31.3	2.0	16.7	20.7
UL 06/2	T	0.4136	0.7215	remained	115.4	192.8	154.1	54.8
	PC	0.4771	1.3913	increased	15.4	92.8	54.1	54.8
UL 07/1	T	0.8509	1.7493	remained	2.9	3.9	3.4	0.7
	PC	0.0251	0.0682	decreased	97.1	96.1	96.6	0.7
UL 07/2	T	0.6457	1.6868	remained	35.2	30.3	32.8	3.5
	PC	0.2275	0.5119	decreased	64.8	69.7	67.2	3.5
UL 12/1	T	0.5756	1.1077	remained	72.5	73.9	73.2	1.0
	PC	0.4173	0.8188	decreased	27.5	26.1	26.8	1.0
UL 12/2	T	0.5656	0.6117	remained	17.1	29.2	23.2	8.6
	PC	0.0968	0.1788	decreased	82.9	70.8	76.8	8.6

Supplementary Table 1B:

ratio		forward	reverse	PC vs. AC	forward [%]	reverse [%]	mean [%]	SD [%]
UL 06/1	PC	0.2264	0.4795	remained	24.8	23.2	24.0	1.1
	AC	0.0561	0.1113	decreased	75.2	76.8	76.0	1.1
UL 06/2	PC	0.4771	1.3913	remained	77.7	81.2	79.4	2.5
	AC	0.3707	1.1297	decreased	22.3	18.8	20.6	2.5
UL 07/1	PC	0.0251	0.0682	remained	46.9	58.8	52.9	8.4
	AC	0.0118	0.0401	decreased	53.1	41.2	47.1	8.4
UL 07/2	PC	0.2275	0.5119	remained	27.0	27.7	27.3	0.5
	AC	0.0613	0.1419	decreased	73.0	72.3	72.7	0.5
UL 12/1	PC	0.4173	0.8188	remained	47.0	32.3	39.7	10.4
	AC1	0.1962	0.2646	decreased	53.0	67.7	60.3	10.4
	PC	0.4173	0.8188	remained	25.1	20.3	22.7	3.3
	AC2	0.1045	0.1665	decreased	74.9	79.7	77.3	3.3
UL 12/2	PC	0.0968	0.1788	remained	69.9	64.4	67.2	3.9
	AC1	0.0677	0.1151	decreased	30.1	35.6	32.8	3.9
	PC	0.0968	0.1788	remained	65.9	79.4	72.6	9.6
	AC2	0.0638	0.1420	decreased	34.1	20.6	27.4	9.6

Supplementary Table 1C:

ratio		forward	reverse	PC vs. P	forward [%]	reverse [%]	mean [%]	SD [%]
UL 06/1	PC	0.2264	0.4795	remained	41.5	45.9	43.7	3.1
	P1	0.0941	0.2202	decreased	58.5	54.1	56.3	3.1
	PC	0.2264	0.4795	remained	2.2	4.2	3.2	1.4
	P2	0.0050	0.0200	decreased	97.8	95.8	96.8	1.4
	PC	0.2264	0.4795	remained	2.2	4.2	3.2	1.4
	P3	0.0050	0.0200	decreased	97.8	95.8	96.8	1.4
UL 06/2	PC	0.4771	1.3913	remained	31.1	27.0	29.1	2.9
	P1	0.1484	0.3762	decreased	68.9	73.0	70.9	2.9
	PC	0.4771	1.3913	remained	17.0	10.5	13.8	4.6
	P2	0.0813	0.1459	decreased	83.0	89.5	86.2	4.6
	PC	0.4771	1.3913	remained	9.4	11.3	10.3	1.3
	P3	0.0449	0.1566	decreased	90.6	88.7	89.7	1.3
UL 07/1	PC	0.0251	0.0682	remained	2.0	1.5	1.7	0.4
	P1	0.0005	0.0010	decreased	98.0	98.5	98.3	0.4
	PC	0.0251	0.0682	remained	2.0	1.5	1.7	0.4
	P2	0.0005	0.0010	decreased	98.0	98.5	98.3	0.4
	PC	0.0251	0.0682	remained	2.0	1.5	1.7	0.4
	P3	0.0005	0.0010	decreased	98.0	98.5	98.3	0.4
UL 07/2	PC	0.2275	0.5119	remained	22.0	19.6	20.8	1.7
	P1	0.0500	0.1003	decreased	78.0	80.4	79.2	1.7
	PC	0.2275	0.5119	remained	5.8	8.7	7.3	2.0
	P2	0.0133	0.0445	decreased	94.2	91.3	92.7	2.0
	PC	0.2275	0.5119	remained	1.3	1.0	1.1	0.2
	P3	0.0030	0.0050	decreased	98.7	99.0	98.9	0.2

Supplementary Table 1D:

ratio		forward	reverse
UL 06/1	SP1	0.4095	1.3227
	SP2	0.2014	0.3694
	SP3	0.0188	0.0547
UL 06/2	SP1	0.4770	1.6032
	SP2	0.3087	0.7184
	SP3	0.1677	0.4250
UL 07/1	SP1	0.0283	0.1057
	SP2	0.0090	0.0362
	SP3	0.0004	0.0010
UL 07/2	SP1	0.5556	1.4147
	SP2	0.2022	0.4354
	SP3	0.0458	0.1267
UL 12/1	S1	0.4850	0.9174
	S2	0.5792	1.1455
	S3	0.4533	0.8581
	S4	0.2590	0.4359
	S5	0.3338	0.7155
UL 12/2	S1	0.3714	0.8249
	S2	0.4066	0.6809
	S3	0.2161	0.4562
	S4	0.2319	0.6153
	S5	0.2962	0.6476

The ratio of mutated to non-mutated peaks of ULs in forward and reverse sequencing direction of tissue (T), primary culture (PC), "aging" primary culture (AC), passages (P1, P2, P3), and supernatants of primary cultures (S1, S2, S3, S4, S5) and passages (SP1, SP2, SP3) are listed by comparison to corresponding basis. The ensuing quotients are displayed as percental remain and decrease for both sequencing directions. Standard deviation (SD) and mean are determined by the percentage of forward and reverse sequencing direction as remain or decrease.
